# Identification of reference genes for circulating long noncoding RNA analysis in serum of cervical cancer patients

**DOI:** 10.1002/2211-5463.12523

**Published:** 2018-09-28

**Authors:** Tawin Iempridee, Suphachai Wiwithaphon, Kitiya Piboonprai, Pornpitra Pratedrat, Phattharachanok Khumkhrong, Deanpen Japrung, Sasithon Temisak, Somsak Laiwejpithaya, Pattama Chaopotong, Tararaj Dharakul

**Affiliations:** ^1^ National Nanotechnology Center National Science and Technology Development Agency Pathum Thani Thailand; ^2^ National Institute of Metrology Pathum Thani Thailand; ^3^ Department of Obstetrics and Gynecology Faculty of Medicine Siriraj Hospital Mahidol University Bangkok Thailand; ^4^ Department of Immunology Faculty of Medicine Siriraj Hospital Mahidol University Bangkok Thailand

**Keywords:** cervical cancer, circulating RNA, diagnostic biomarker, long noncoding RNA, reference genes, serum

## Abstract

Circulating lncRNAs have attracted considerable attention as potential noninvasive biomarkers for diagnosing cancers. RT‐qPCR is the canonical technique for detecting circulating RNA and depends largely on stable reference genes for data normalization. However, no systematic evaluation of reference genes for serum lncRNA has been reported for cervical cancer. Here, we profiled and validated lncRNA expression from serum of cervical cancer patients and controls using microarrays and RT‐qPCR. We identified lncRNA RP11‐204K16.1, XLOC_012542, and U6 small nuclear RNA as the most stable reference genes based on geNorm, NormFinder, BestKeeper, delta Ct, and RefFinder. These genes were suitable also for samples from different age groups or with hemolysis. Additionally, we discovered lncRNA AC017078.1 and XLOC_011152 as candidate biomarkers, whose expression was down‐regulated in cervical cancer. Our findings could aid research on circulating lncRNA and the discovery of blood‐based biomarkers for cervical cancer diagnosis.

**Abbreviations**
AUC: area under the ROC curve
cfRNA: cell‐free RNA
CIN3: cervical squamous intraepithelial neoplasia 3
Cq: cycle of quantification
cRNA: complementary RNA
GAPDH: glyceraldehyde‐3‐phosphate dehydrogenase
lncRNA: long noncoding RNA
miRNA: microRNA
OD: optical density
ROC: receiver operating characteristic
RT‐qPCR: reverse transcription‐quantitative polymerase chain reaction
SFM: serum‐free medium

Cervical cancer is one of the most common gynecological cancers in low‐ and middle‐income countries, contributing to 10–15% of cancer death in females worldwide 1. Although it has helped decrease incidence and mortality, the Pap screening test can be inconclusive and generates a relatively high percentage of false negatives 2. Furthermore, because the method requires a vaginal examination, participation of Thai women in cervical cancer screening programs remains low 3. The availability of minimally invasive tests may improve women's participation, thereby reducing the burden of cervical cancer. However, developing new screening options requires yet‐to‐be‐identified circulating biomarkers.

Long noncoding RNA (lncRNA) comprise noncoding RNA of >200 nucleotides in length, which are involved in a wide range of physiological and pathological processes including tumorigenesis 4, 5. Importantly, certain lncRNAs are stable and detectable in body fluids such as plasma, serum, and urine, making them of interest for the development of minimally invasive tests 5, 6. Recently, lncRNAs HOTAIR and PVT1 have been proposed as serum biomarkers for cervical cancer 5, 7. These initial findings highlight the need for further explorations of circulating lncRNA as cervical cancer biomarkers. To do so, it is indispensable to accurately determine the expression of circulating lncRNA present at extremely low levels 8.

Reverse transcription‐quantitative polymerase chain reaction (RT‐qPCR) is frequently used to quantify circulating RNA 6. However, its accuracy depends greatly on normalization to reference genes stably expressed in the investigated samples. Studies on cervical cancer continue to use common reference genes including glyceraldehyde‐3‐phosphate dehydrogenase (GAPDH) 7, 9 and U6 5 as circulating RNA reference genes without systemically evaluating their suitability. The use of circulating microRNA (miRNA) reference genes is not ideal for long RNA expression analysis as sample preparation steps including first‐strand cDNA synthesis are usually different, hampering the detection of lncRNA and miRNA from the same cDNA preparation. All of this may lead to data misinterpretation and constitutes an unmet challenge.

In the present study, we attempted to identify circulating RNA reference genes for lncRNA analysis from serum of cervical cancer patients and healthy controls using microarrays and RT‐qPCR. Candidate biomarkers were analyzed for expression stability using statistical algorithms. The stability of optimal reference genes was compared to the miRNA reference genes and was evaluated in samples from different age groups and with hemolysis. Finally, we explored potential serum lncRNA biomarkers capable of discriminating cervical cancer patients from controls and determined their expression levels in both cervical cancer and normal cells.

## Materials and methods

### Serum sample collection

Collection of serum samples was approved by the Siriraj Institutional Review Board, Faculty of Medicine Siriraj Hospital, Mahidol University (Si 474/2015), in accordance with the Helsinki Declaration. All participants signed a written consent form prior to sample collection. Serum of cervical cancer patients (*n *= 36) and a patient with cervical squamous intraepithelial neoplasia 3 (CIN3) was collected at the tumor clinic, Department of Obstetrics and Gynecology, Faculty of Medicine Siriraj Hospital. The controls (*n *= 31) were recruited from volunteers without evidence of cancer and with a normal Pap test within the previous year. Peripheral blood was obtained by venous puncture. Blood was clotted for 30 min and centrifuged at 3000 ***g*** for 15 min at 4 °C. Serum was withdrawn without disturbing the buffy coat and stored at −80 °C.

### Cell culture

Human primary fibroblasts (CRL‐2708), HPV‐16 E6/E7‐transformed ectocervical Ect1/E6E7 (CRL‐2614), HPV‐positive cervical carcinoma [HeLa (CCL2), SiHA (HTB‐35), and MS751 (HTB‐34)], and HPV‐negative cervical carcinoma C33a (HTB‐31) cell lines were purchased from the American Type Culture Collection (Manassas, VA, USA). Primary fibroblasts, HeLa, SiHa, MS751, and C33a cells were cultured in Dulbecco's modified Eagle medium (HyClone, Thermo Fisher Scientific, Waltham, MA, USA) supplemented with 10% fetal bovine serum (HyClone) and 100 units·mL^−1^ penicillin plus 100 μg·mL^−1^ streptomycin. The Ect1/E6E7 cell line was grown in keratinocyte serum‐free medium (SFM) with Keratinocyte‐SFM supplement (Gibco, Thermo Fisher Scientific, Waltham, MA, USA). Cells were maintained at 37 °C in a 5% CO_2_ incubator.

### RNA extraction, quantification, and quality assessment

Total RNA was extracted from 800 μL of serum samples using the Plasma/Serum Circulating and Exosomal RNA Purification Kit (Norgen Biotek, Thorold, ON, Canada). Serum RNA concentration was quantified by the Fragment Analyzer™ Automated CE System using the High Sensitivity RNA Analysis Kit (AATI, Ankeny, IA, USA); fragment size and concentration were determined by prosize ^®^ software (AATI). Samples with RNA concentration below 0.27 ng·μL^−1^ were concentrated by an RNA cleanup and concentration kit (Norgen Biotek).

A qPCR quality control based on detection of three commonly found serum/plasma miRNAs, let‐7d, let‐7g, and let‐7i 10, 11, and GAPDH mRNA was employed to ensure that the quality of serum RNA was not compromised by the presence of inhibitors affecting cDNA synthesis and/or qPCR products. Expression of let‐7d, let‐7g, let‐7i, and GAPDH was detected in all RNA samples.

To isolate total RNA from cell lines, RNA was extracted using the RNeasy mini kit (Qiagen, Holden, Germany). RNA samples were quantified on a NanoDrop One UV‐Vis Spectrophotometer (Thermo Scientific). All cellular RNA samples exhibited optical density (OD) A260/A280 and OD A260/A230 ratios of 1.8–2.1.

### Microarray and data analysis

For microarray analysis, total RNA from serum was amplified and transcribed into fluorescent complementary RNA (cRNA) using the manufacturer's Agilent's Quick Amp Labeling protocol (version 5.7, Agilent Technologies, Santa Clara, CA, USA). The cRNA were hybridized onto the LncPath™ Human Cancer Array (8*15K, Arraystar, Rockville, MD, USA). After washing the slides, the arrays were scanned with the G2505C Agilent Scanner. Images were analyzed by Agilent Feature Extraction software. Quantile normalization and data processing were performed in R. The microarray work was performed by Arraystar.

### RT‐qPCR and target‐specific pre‐amplification

To detect lncRNA and mRNA, total RNA (4 ng for serum RNA or 1.5 μg for RNA from cell lines) was converted to cDNA using the iScript Advanced cDNA Synthesis Kit (Bio‐Rad, Hercules, CA, USA). The synthesized cDNA was diluted twofold, and 2 μL of each cDNA was used as a template for qPCR.

For pre‐amplification of reference lncRNA from serum cDNA, a multiplex pre‐amplification reaction was performed using 2× SsoAdvanced™ PreAmp Supermix (Bio‐Rad), primer pool (0.5 μm each), and 5 μL of cDNA template. The reaction was activated at 95 °C for 3 min, followed by 10 cycles at 95 °C for 15 s and 58 °C for 4 min. The pre‐amplified product was diluted 10‐fold and stored at −20 °C for qPCR analysis.

QPCR products were performed in a Bio‐Rad CFX96 Touch using a SsoAdvanced™ Universal SYBR Green Supermix (Bio‐Rad). PCR conditions were as follows: 98 °C for 30 s, 40 cycles of 98 °C for 5 s, and 58.8–60 °C for 30 s, followed by melting curve analysis, from 65 to 96 °C with increments of 0.5 °C per cycle. Based on the slopes of standard curves, all qPCR primers exhibited amplification efficiencies of 93.51–99.17% (Table S1). Primer sequences and annealing temperatures (*T*_a_) are listed in Table S1.

To detect miRNA, 1 ng of total RNA was polyadenylated, ligated to the 5′ end adaptor, converted to cDNA, and amplified using the TaqMan™ Advanced miRNA cDNA Synthesis Kit (Thermo Fisher Scientific). Expression levels of target miRNAs were quantified by qPCR using TaqMan^®^ Advanced miRNA Assays (Thermo Fisher Scientific). Two technical replicates were performed for each sample, and the average cycle of quantification (Cq) values was calculated using Bio‐Rad cfx software. Cq values for all samples were at least 5 Cq below a no‐template control 12. Reactions with a Cq value above 35 were considered below detection limit and were excluded from analysis 13.

### Analysis of reference gene expression stability

The expression stability of reference genes was determined using widely used statistical algorithms, including geNorm 14, NormFinder 15, BestKeeper 16, and delta Ct method 17 integrated within the RefFinder online tool (http://leonxie.esy.es/RefFinder/). The ranking of gene stability was generated by each algorithm, and the overall final ranking of candidate reference genes was determined by RefFinder 18.

### Statistical analysis

Box plots were generated by BoxPlotR 19, and statistical analysis was performed using graphpad instat version 3. The Mann–Whitney nonparametric *U*‐test was used for two‐group comparisons, whereas the Kruskal–Wallis nonparametric test with Dunn's post‐test was used to compare three or more groups. One‐way ANOVA with Tukey HSD was used to compare gene expression among cell lines. Receiver operating characteristic (ROC) curves and area under the ROC curve (AUC) were generated by MedCalc. A *P* value <0.05 was considered statistically significant.

## Results and Discussion

### Analysis of circulating RNA from serum samples

Circulating cell‐free RNA (cfRNA) in serum, plasma, and other body fluids harbors great potential in minimally invasive diagnosis and prognosis. Nevertheless, cfRNA usually exists in very low amounts, below the detection limit of standard spectrophotometric methods 20, 21. Several studies have normalized the amount of RNA based on sample volume or the spike‐in RNA added in the lysis buffer 22, 23. However, these methods cannot compensate for variations in the amount of extracted RNA inherent to a biological sample. To ameliorate this problem, we quantified total RNA using a system that allows for size separation and has an RNA detection limit of 50 pg·μL^−1^.

**Figure 1:**
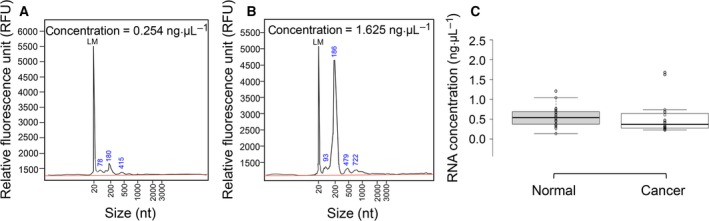
Size distribution and concentrations of total RNA extracted from serum of normal and cervical cancer patients. (A, B) Postseparation electropherograms of serum RNA from cervical cancer patients with relatively low (A) and high (B) RNA concentrations. LM represents a 15‐nt marker. (C) Box plot displaying the range of total RNA concentrations obtained from serum of normal (*n *=* *21) and cervical cancer patients (*n *=* *20). Box limits indicate the 25th and 75th percentiles with median on the center lines and data points plotted as open circles.

As shown in Figs 1A and 1B, cfRNA typically contains a broad range of RNA sizes, ranging from 20 nt to 1 kb, with the peak at 15 nt representing the low molecular weight marker and another distinct peak at 170–190 nt. The total concentration of extracted cfRNA varied greatly across samples (see examples in Fig. 1A vs 1B), reinforcing the need for accurate determinations. Importantly, there was no significant difference in the average amount of serum RNA between cervical cancer and control groups (Fig. 1C), suggesting that normalization based on the amount of RNA input will not generate bias associated with differential cfRNA quantity between the two groups.

cfRNA from body fluids is often found in short fragments (<1000 nt) 24 and usually lacks ribosomal bands. Thus, the RNA integrity number cannot be reliably used to assess cfRNA quality. In addition, as the concentration of cfRNA is extremely low, its A260/A230 and A260/A280 ratios often fall outside the range generally accepted as indicating a pure RNA sample 25. To overcome these limitations, we determined the quality of serum RNA based on the ability to detect three miRNA, let‐7d, let‐7g, and let‐7i, and GAPDH mRNA, known to be expressed in serum/plasma 5, 10, 11, 26. As shown in Fig. S1, expression of both miRNA and mRNA controls was detected in all samples, indicating that all RNA samples were of adequate quality for efficient amplification via RT‐qPCR.

As cfRNA yields from plasma or serum are very low and highly variable across samples, total RNA concentration should be determined using an ultrasensitive method to ensure that the same amount of RNA input is used for downstream applications. Even though circulating RNAs are inherently unable of meeting conventional RNA quality standards 25, a qPCR‐based method should be applied to ensure high‐quality data from subsequent gene expression analysis.

### Screening for candidate reference genes for circulating lncRNA analysis

To screen for potential reference genes, total RNA from six normal and six cervical cancer samples (Table 1) was amplified and transcribed into cRNA using a random priming method. Expression analysis comprised 2829 lncRNA and 1906 potential coding targets (Fig. S2). To identify potential reference genes, lncRNA expression was analyzed for relative standard deviation across samples and expression levels. As shown in Fig. S3, we selected lncRNA AF015262.2 (R3) and RP4‐609E1.2 (R4) as candidate reference genes since they were highly and stably expressed across samples. In addition, we chose lncRNAs RP11‐204K16.1 (R1) and XLOC_012542 (R2) as their expression was stable based on RT‐qPCR analysis, even though they were originally identified by microarray as significantly down‐ and up‐regulated, respectively, in cervical cancer patients (data not shown). Discrepancies in gene expression data between microarray and RT‐qPCR platforms have been documented previously, especially with regard to poorly abundant transcripts such as circulating RNA 27, 28, 29. GAPDH and U6 were also included in the study due to their previous use as reference genes for serum RNA 5, 7, 9.

**Table 1:** Characteristics of patients and controls for reference gene expression analysis

Sample set	LncRNA microarray^a^		RT‐qPCR	
Characteristics	Cervical cancer	Control	Cervical cancer	Control
Healthy control	–	6 (100%)	–	26 (100%)
Cervical cancer	6 (100%)	–	24 (100%)	–
Age (mean ± SD)	49.67 ± 1.63	50.17 ± 1.17	50.83 ± 6.96	49.96 ± 5.10
Histology				
Adenocarcinoma	2 (33.3%)	–	11 (45.8%)	–
Squamous	4 (66.7%)	–	13 (54.2%)	–
FIGO stage				
IB1	3 (50%)	–	6 (25%)	–
IIA1	–	–	1 (4.2%)	–
IIA2	–	–	1 (4.2%)	–
IIB	–	–	4 (16.7%)	–
IIIB	2 (33.3%)	–	10 (41.7%)	–
At least IIIB	–	–	1 (4.2%)	–
IVA	1 (16.7%)	–	1 (4.2%)	–

a Three cervical cancer and four control samples in microarray experiments were also used in the validation set by RT‐qPCR.

**Figure 2:**
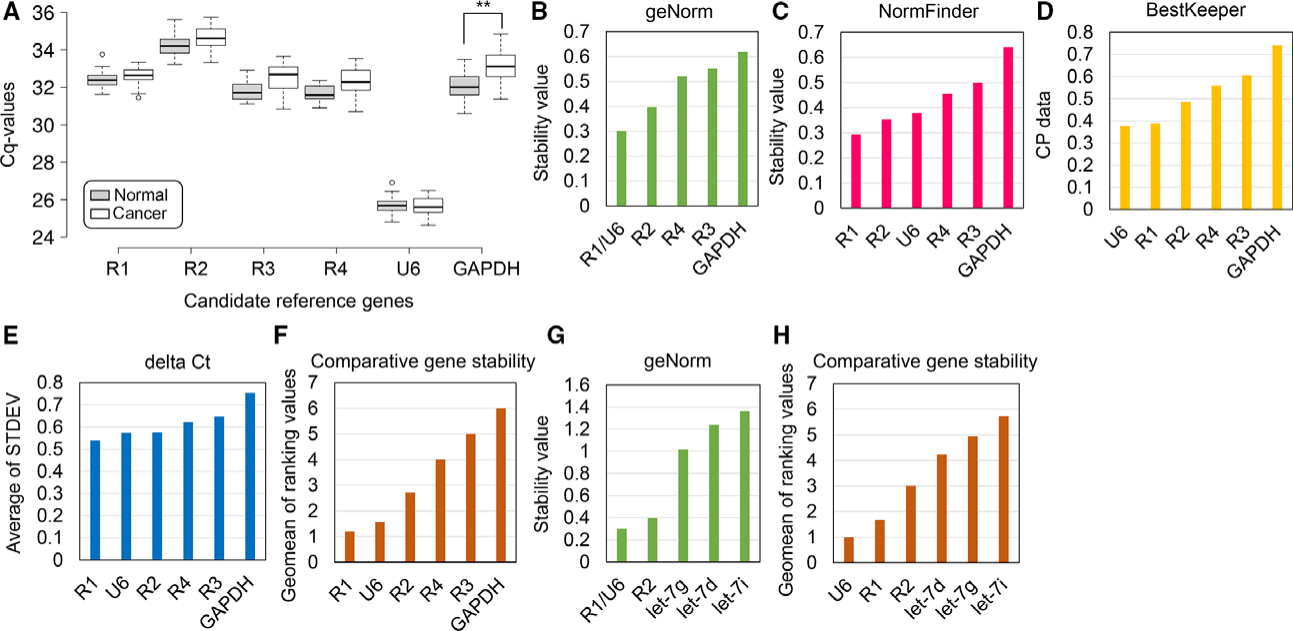
Expression levels and stability of candidate reference genes in serum of cervical cancer patients and controls. (A) Box plots showing Cq values of reference genes in serum from normal (*n* = 26) and cervical cancer patients (*n* = 24). Center lines show the medians, box limits indicate the 25th and 75th percentiles, whiskers extend 1.5 times the interquartile range from the 25th and 75th percentiles, and outliers are represented by dots. Groups were compared using Mann–Whitney nonparametric U‐test (**P <* *0.01 compared to the normal group). (B–F) LncRNA expression stability ranking of candidate reference genes according to geNorm (B), NormFinder (C), BestKeeper (D), delta Ct (E), or comprehensive gene stability by RefFinder (F). (G, H) Expression stability of reference genes R1, R2, and U6 compared to miRNA reference genes let‐7d, let‐7g, and let‐7i as determined by geNorm (G) or RefFinder (H).

To validate candidate reference genes, we utilized RT‐qPCR to quantify their expression levels from 24 cervical cancer patients (12 stage I/II and 12 stage III/IV) and 26 age‐matched controls (Table 1). As shown in Fig. 2A, the average Cq values were 32.5, 34.4, 32.1, 32.0, 25.7, and 32.6 for R1, R2, R3, R4, U6, and GAPDH, respectively. Although expression levels of circulating lncRNA are extremely low 8, we did not employ the pre‐amplification step prior to qPCR to avoid introducing bias caused by uneven PCR amplification 30.

Next, we compared the expression profiles of reference genes between normal and cancer sample sets. Cq values differed significantly between the two groups only for GAPDH but not the other candidate genes, indicating that GAPDH was not stably expressed and was not a suitable reference gene (Fig. 2A).

### Expression stability of candidate reference lncRNA

Next, we evaluated expression stability of candidate reference genes based on five algorithms. GeNorm assesses the stability measure (*M*) of each gene, with *M* < 0.5 generally considered stably expressed 31. Here, the *M*‐values of U6, R1, and R2 were <0.5, with R1 and U6 being ranked as the optimal combination of reference genes (Fig. 2B).

NormFinder ranks the order of genes based on intra‐ and intergroup variations with a lower value representing a higher stability 15. As shown in Fig. 2C, the rank order of the candidate reference genes from the highest to lowest stability was as follows: R1, R2, U6, R4, R3, and GAPDH, respectively. Notably, the top three reference genes, R1, U6, and R2, were recommended by both geNorm and NormFinder.

BestKeeper analyzes gene stability based on standard deviation (SD), with a lower SD representing a more stable expression 16. As shown in Fig. 2D, U6 was ranked as the most stable gene, followed by R1, R2, R4, R3, and GAPDH, respectively. Finally, according to the delta Ct method, R1 was the most stably expressed genes, followed by U6, R2, R4, R3, and GAPDH, respectively (Fig. 2E). Thus, all four algorithms consistently picked R1, U6, and R2 as the three most stable reference genes.

Next, we utilized RefFinder to generate an overall ranking of candidate reference genes 18. R1 was ranked as the most stable gene followed by U6 and R2, whereas GAPDH was the least stably expressed gene (Fig. 2F). Taken together, we conclude that R1, U6, and R2 are suitable reference genes for serum samples from both cervical cancer patients and controls.

### Expression stability of candidate lncRNA and miRNA reference genes

Next, we compared expression stability of candidate reference genes R1, U6, and R2 with miRNAs let‐7d, let‐7g, and let‐7i, previously reported as the most stable reference genes for normalizing serum miRNAs 11. Serum RNA was converted to cDNA, amplified, and quantified. As shown in Figs 2G and 2H, both geNorm and RefFinder indicated that R1 and U6 were the most stably expressed genes, followed by R2, let‐7d/let‐7g, and let‐7i, respectively. Accordingly, R1, U6, and R2 are statistically superior to miRNAs let‐7d, let‐7g, and let‐7i for circulating RNA expression studies in cervical cancer patients.

### Expression stability of candidate reference genes in serum from different age groups

**Figure 3:**
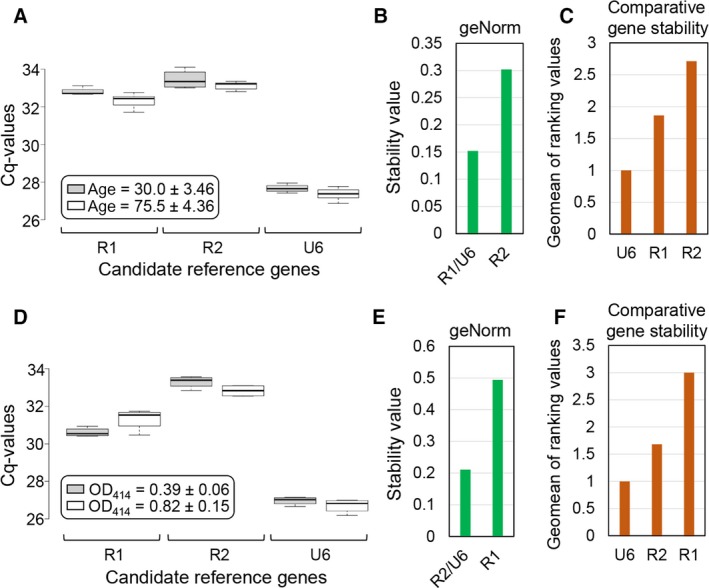
Expression stability of candidate reference genes in serum from different age groups and serum with hemolysis. (A, D) Box plots of Cq values for candidate reference genes in serum from two age groups, 30 ± 3.46 years (*n* = 4) or 75.5 ± 4.36 years (*n* = 5) (A), and from samples with no/low or severe hemolysis (*n* = 4) (D). Center lines show the medians, box limits indicate the 25th and 75th percentiles, and whiskers extend 1.5 times the interquartile range from the 25th and 75th percentiles. (B–C, E–F) Reference gene stability ranked according to geNorm (B, E) and RefFinder (C, F).

To further validate the stability of the top three reference genes, we investigated their expression levels in serum derived from cervical cancer patients of various ages (Table 2). Cq values of all candidate genes were similar between the young (30 years old; *n* = 4) and the old (75 years old; *n* = 5) cohorts (Fig. 3A), indicating stable expression between the two groups. Ranking by geNorm showed that all reference genes were stably expressed (*M* < 0.5), with R1 and U6 being the most stable (Fig. 3B). RefFinder ranked U6 as the most stable gene followed by R1 and R2, respectively (Fig. 3C). These results indicate that U6, R1, and R2 are suitable reference genes for analysis of serum from different age groups, with U6 and R1 as the most stable combination.

**Table 2:** Characteristics of subjects used for investigating effects of age and hemolysis on the expression of candidate reference genes

Characteristics	Young group	Old group	Non/slightly hemolyzed and severely hemolyzed group
Healthy control	–	–	3 (75%)
Precancer	–	–	1 (25%)
Cervical cancer	4 (100%)	5 (100%)	–
Age (mean ± SD)	30.0 ± 3.46	75.5 ± 4.36	38.75 ± 7.85
Histology			
Adenocarcinoma	–	–	–
Squamous	4 (100%)	5 (100%)	–
FIGO stage			
CIN3	–	–	1 (25%)
IB1	2 (50%)	–	–
IIB	1 (25%)	–	–
IIIA	–	1 (20%)	–
IIIB	1 (25%)	4 (80%)	–

### Expression stability of candidate reference genes in samples with no/low or severe hemolysis

Hemolysis affects approximately 43% of clinical specimens as determined by free hemoglobin >0.5 g·L^−1^ or about 6% based on visual detection by pink/red discoloration 32, 33. Importantly, hemolysis can greatly alter the level of serum miRNA due to release of miRNA in blood cells 34. To investigate the effect of hemolysis on expression levels of candidate reference genes, we selected noncancer samples (*n* = 4, Table 2) containing fractions with both no/low level and severe hemolysis. The hemolyzed fractions exhibited red discoloration and high absorbance at 414 nm (OD_414_ = 0.8), indicating the presence of free hemoglobin 10, whereas fractions with no/low hemolysis did not show any pink or red coloration and exhibited OD_414_ <0.4.

As shown in Fig. 3D, Cq values for R1, R2, and U6 were not significantly different between normal and cervical cancer sets, indicating that they were stably expressed in the two groups. Analysis by geNorm indicated that all reference genes exhibited *M*‐values <0.5, with R2 and U6 being the most stable (Fig. 3E). RefFinder ranked U6 as the most stable gene, followed by R2 and R1, respectively (Fig. 3F). Therefore, U6, R1, and R2 are suitable reference genes for analysis of serum with or without hemolysis, and with U6 and R2 as the most optimal combination.

### Pre‐amplification of candidate lncRNA reference genes

MiRNA constitutes the majority (40–46%) of circulating extracellular RNA, whereas lncRNA accounts for only 2–10% of all RNA species 35, 36. Due to its low abundance, highly sensitive pre‐amplification qPCR methods are usually required to detect circulating lncRNA 8, 24. To test whether such strategy improved the sensitivity of lncRNA reference gene detection, we carried out target‐specific pre‐amplification of R1, R2, R3, and R4 from a serum cDNA using the same primer set (Table S1). As shown in Fig. S4, pre‐amplification caused Cq values of all reference lncRNAs to decrease by approximately five cycles, from 30.8–32.3 to 26.3–27.1, indicating successful enrichment of the targets. Therefore, we conclude that a target‐specific pre‐amplification procedure could be used to overcome the scarcity of serum lncRNAs and improve the utility of identified reference genes for future circulating lncRNA studies.

### Identification of candidate lncRNA biomarkers from serum samples

**Figure 4:**
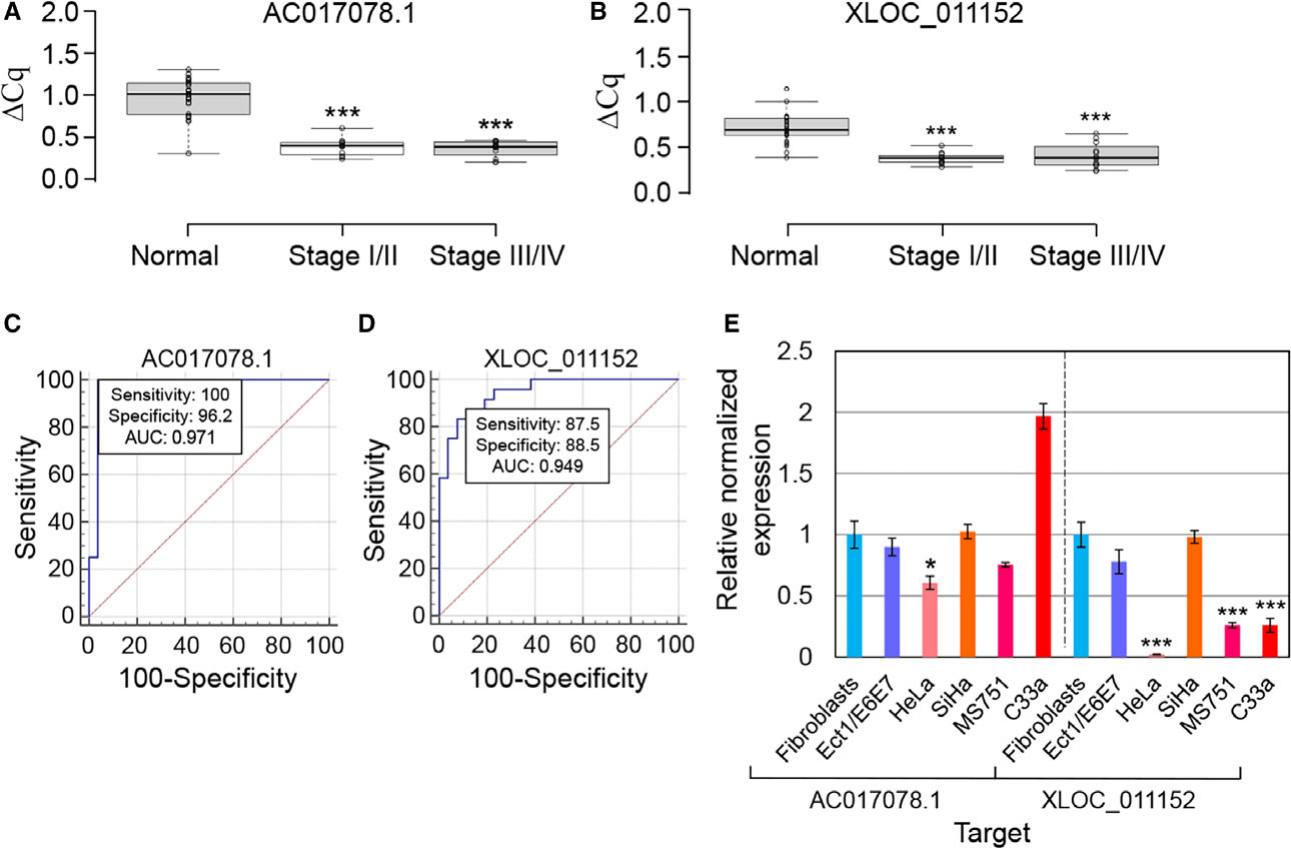
Expression levels of candidate lncRNA biomarkers in clinical samples, cell lines, and analysis of their diagnostic performances. (A, B) Box plots showing differential expression of lncRNAs AC017078.1 (A) and XLOC_011152 (B) in normal (*n* = 26), cervical cancer stages I/II (*n* = 12), and stages III/IV (*n* = 12). Groups were compared using nonparametric Kruskal–Wallis test with Dunn's post‐test (***P <* *0.001 compared to the normal group). (C, D) Receiver operating characteristic curves for lncRNAs AC017078.1 (C) and XLOC_011152 (D) to discriminate cervical cancer patients (*n* = 24) from controls (*n* = 26). AUC: area under the curve. (E) Relative levels of lncRNA biomarkers in primary fibroblasts, immortalized ectocervical cell line (Ect1/E6E7), and four cervical cancer cell lines. RT‐qPCR values were normalized internally to GAPDH and RPS13 and externally to the expression level in primary fibroblasts. One‐way ANOVA with Tukey HSD was used to compare gene expression between each cell line and fibroblasts. Bars indicate means ± 1 SEM; assays were performed in triplicate. *P <* *0.05; ***P <* *0.001.

Finally, we attempted to identify serum lncRNAs differentially expressed in cervical cancer patients relative to controls using a combination of R1 and U6 as reference genes. RT‐qPCR revealed that lncRNAs AC017078.1 (RPL26P15) and XLOC_011152 (lnc‐GPR132‐1) were significantly down‐regulated in stage I/II (*n* = 12) and III/IV (*n* = 12) cancers compared to the control group (*n* = 26) (Figs 4A and 4B), suggesting that they could potentially serve as circulating biomarkers for cervical cancer.

Next, we conducted ROC curve analysis to evaluate the diagnostic value for discriminating between cervical cancer (*n* = 24) patients and controls (*n* = 26). As shown in Figs 4C and 4D, the AUC value of lncRNA AC017078.1 was 0.971 (*P *<* *0.0001) and that of lncRNA XLOC_011152 was 0.949 (*P *<* *0.0001). Thus, both serum lncRNA are excellent at separating cervical cancer patients from controls.

To investigate whether expression of these lncRNA biomarkers was down‐regulated also in cervical cancer cells, we next determined the relative expression of lncRNAs AC017078.1 and XLOC_011152 in multiple cervical cancer cell lines, including an immortalized ectocervical cell line (Ect1/E6E7) and primary fibroblasts as controls. As shown in Fig. 4E, comparing to primary fibroblasts, lncRNA AC017078.1 was down‐regulated in HeLa cells, whereas lncRNA XLOC_011152 was down‐regulated in HeLa, MS751, and C33a cell lines. Owing to their down‐regulation in both cervical cancer serum and cell lines, we hypothesize that these lncRNAs play an inhibitory role in cervical cancer progression.

LncRNA AC017078.1 is a processed pseudogene that overlaps in antisense orientation with the intron of protein kinase C‐ε, a protein overexpressed in multiple cancers, which regulates cell transformation, survival, proliferation, and metastasis 37. LncRNA XLOC_011152 is located next to the protein‐coding gene GPR132, a pH‐sensing G protein‐coupled receptor that regulates cancer cell proliferation and metastasis 38. Whether these lncRNAs affect expression of genes in the vicinity and how they contribute to tumorigenesis awaits further investigation. At present, serum lncRNAs AC017078.1 and XLOC_011152 appear suitable biomarkers for cervical cancer diagnosis.

In summary, our results show that lncRNAs RP11‐204K16.1, XLOC_012542, and small RNA U6 are optimal reference genes for serum lncRNA analysis in cervical cancer patients and controls. They are more stably expressed than previously reported miRNA reference genes and could be used with samples from different age groups and with/without hemolysis. In addition, we identified lncRNAs AC017078.1 and XLOC_011152 as potential serum biomarkers with good diagnostic potential for cervical cancer. Although further studies with larger cohorts are required to validate these data, our findings could contribute to the development of minimally invasive diagnostic tests for cervical cancer.

## Conflict of interest

National Science and Technology Development Agency has filed Thai patent applications on behalf of TI, DJ, PP, and ST claiming some of the concepts contemplated in this publication (application numbers 1701001259 and 1701001260).

## Author contributions

TI supervised the study, designed and performed experiments, analyzed data, and wrote the manuscript. SW, KP, PP, and PK performed experiments. DJ and ST analyzed data and provided suggestions. SL and PC collected samples and analyzed clinical data. TD supervised the study. All authors approved the contents of this manuscript.

## Supporting information


**Fig. S1**. qPCR‐based RNA quality control showing expression levels of let‐7d, let‐7g, let‐7i miRNAs, and GAPDH mRNA in serum of cervical cancer patients and controls.
**Fig. S2**. Scatter‐ and volcano‐plots of lncRNA expression profile from LncPath™ Cancer Microarrays. (A) Scatterplot of lncRNAs showing normalized signal for cervical cancer *vs* control. The top green line represents fold change ≥2 (up‐regulated) and the bottom green line indicates fold‐change ≤ −2 (down‐regulated). (B) Volcano plot of lncRNAs statistical significance and fold change. The vertical lines represent 2.0‐fold up and down, respectively, and the horizontal line indicates a *P*‐value of 0.05. The red squares show differentially expressed lncRNAs between cervical cancer *vs* control.
**Fig. S3**. Scatterplot showing lncRNA expression variation across samples and mean expression level from LncPath™ Cancer Microarrays. LncRNAs AF015262.2 (R3) and RP4‐609E1.2 (R4) are indicated.
**Fig. S4**. Target pre‐amplification of candidate lncRNA reference genes. Graphs show expression levels (Cq values) of R1, R2, R3, and R4 reference genes in a serum cDNA before and after pre‐amplification.
**Table S1.** Primers used for qPCR.
