# Supplementary material for: Identification of reference genes for circulating long noncoding RNA analysis in serum of cervical cancer patients
Source: FEBS Open Bio. 2018 Sep 28;8(11):1844–54. doi: 10.1002/2211-5463.12523 (PMC6212646; doi:10.1002/2211-5463.12523)
Supplement: Supplementary file 1 — Fig. S1. qPCR‐based RNA quality control showing expression levels of let‐7d, let‐7g, let‐7i miRNAs, and GAPDH mRNA in serum of cervical cancer patients and controls. Fig. S2. Scatter‐ and volcano‐plots of lncRNA expression profile from LncPath™ Cancer Microarrays. (A) Scatterplot of lncRNAs showing normalized signal for cervical cancer vs control. The top green line represents fold change ≥2 (up‐regulated) and the bottom green line indicates fold‐change ≤ −2 (down‐regulated). (B) Volcano plot of lncRNAs statistical significance and fold change. The vertical lines represent 2.0‐fold up and down, respectively, and the horizontal line indicates a P‐value of 0.05. The red squares show differentially expressed lncRNAs between cervical cancer vs control. Fig. S3. Scatterplot showing lncRNA expression variation across samples and mean expression level from LncPath™ Cancer Microarrays. LncRNAs AF015262.2 (R3) and RP4‐609E1.2 (R4) are indicated. Fig. S4. Target pre‐amplification of candidate lncRNA reference genes. Graphs show expression levels (Cq values) of R1, R2, R3, and R4 reference genes in a serum cDNA before and after pre‐amplification. Table S1. Primers used for qPCR. [file FEB4-8-1844-s001.docx]

**Supporting information**

**Supplementary figure 1**

**
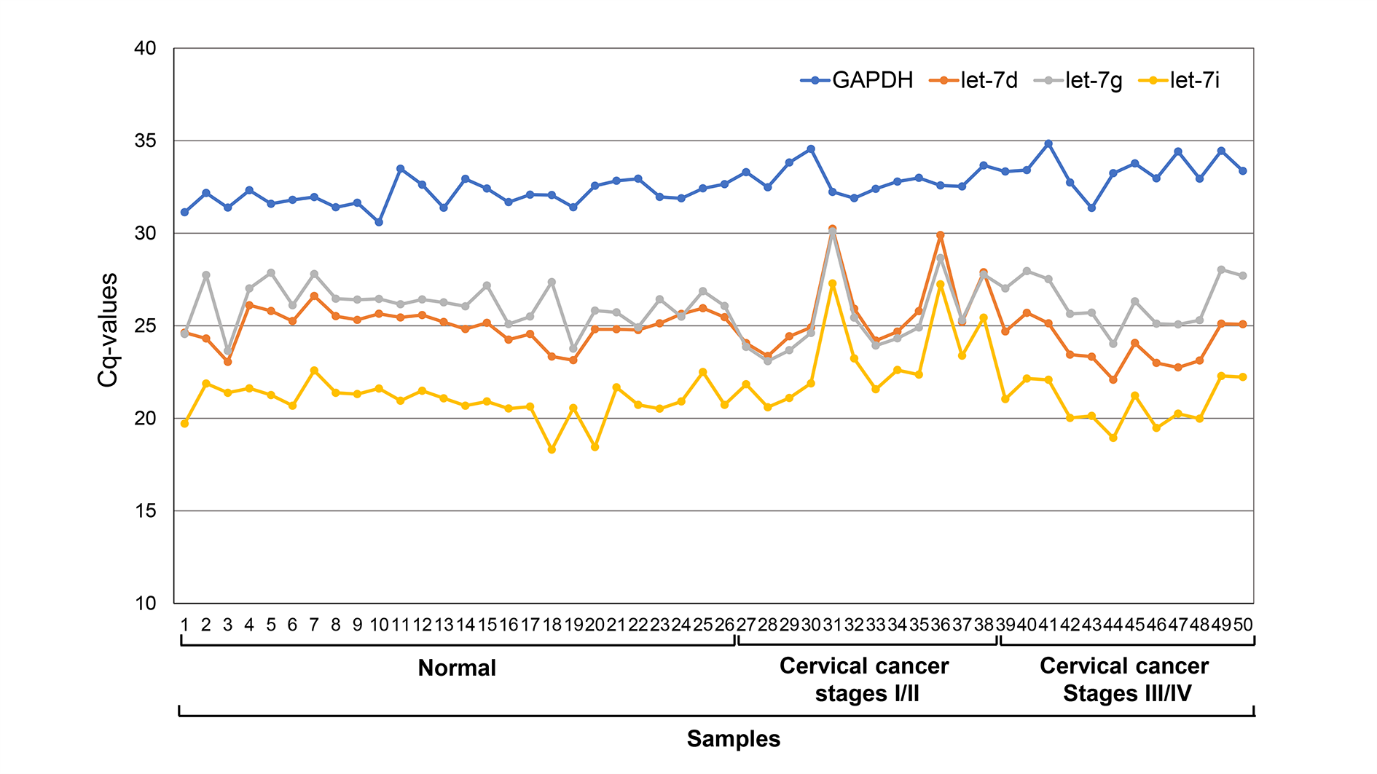
**

**Figure S1**. qPCR-based RNA quality control showing expression levels of let-7d, let-7g, let-7i miRNAs, and GAPDH mRNA in serum of cervical cancer patients and controls.

**Supplementary figure 2**


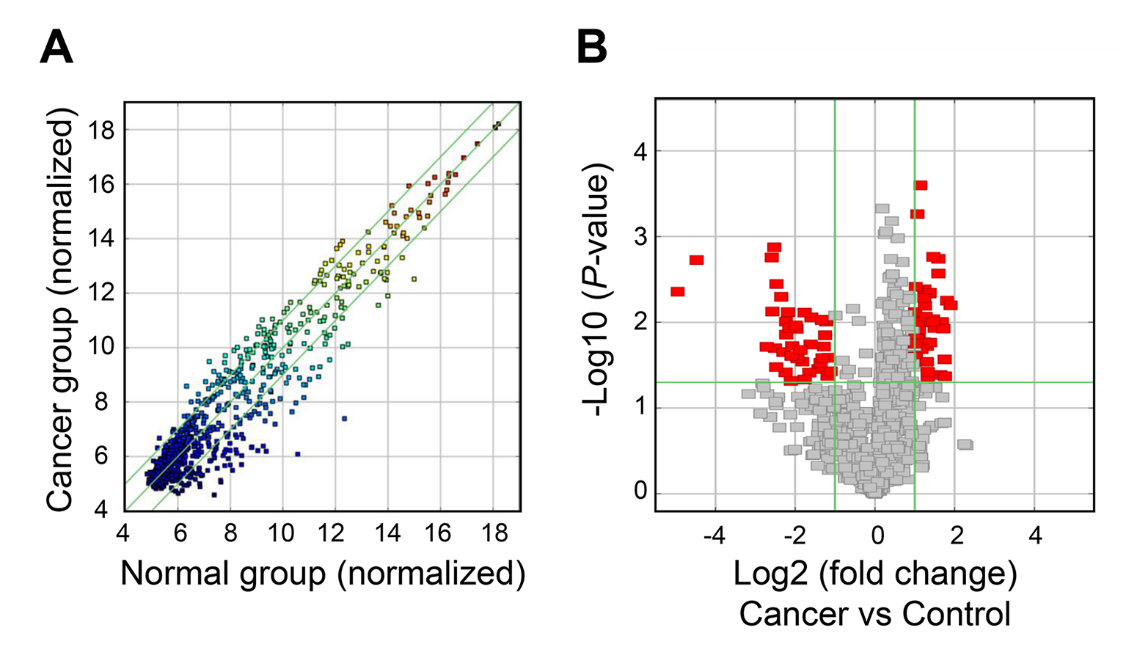


**Figure S2**. Scatter- and volcano-plots of lncRNA expression profile from LncPath™ Cancer Microarrays. (A) Scatterplot of lncRNAs showing normalized signal for cervical cancer *vs* control. The top green line represents fold change ≥ 2 (up-regulated) and the bottom green line indicates fold-change ≤ -2 (down-regulated). (B) Volcano plot of lncRNAs statistical significance and fold change. The vertical lines represent 2.0-fold up and down, respectively, and the horizontal line indicates a *P*-value of 0.05. The red squares show differentially expressed lncRNAs between cervical cancer *vs* control.

**Supplementary figure 3**


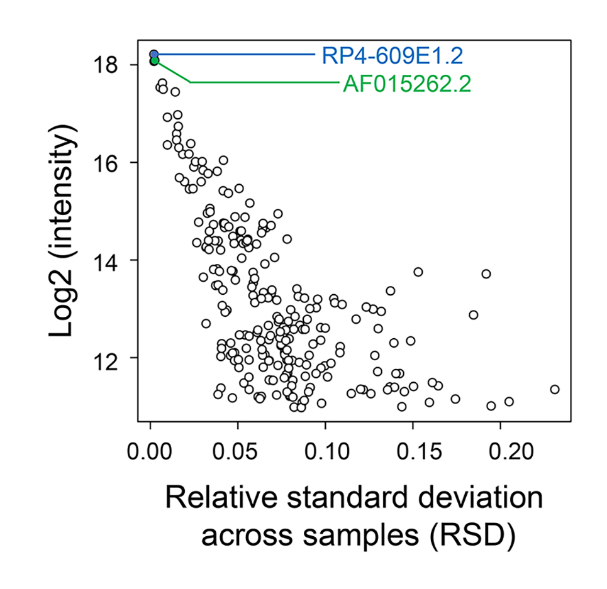


**Figure S3**. Scatterplot showing lncRNA expression variation across samples and mean expression level from LncPath™ Cancer Microarrays. LncRNAs AF015262.2 (R3) and RP4-609E1.2 (R4) are indicated.

**Supplementary figure 4**


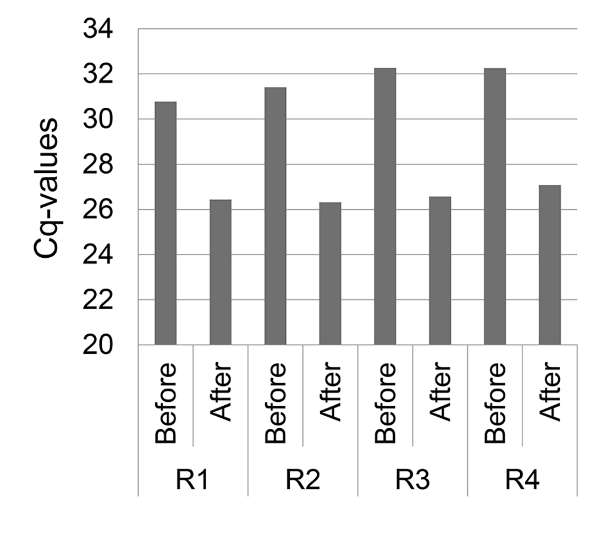


**Figure S4**. Target pre-amplification of candidate lncRNA reference genes. Graphs show expression levels (Cq values) of R1, R2, R3, and R4 reference genes in a serum cDNA before and after pre-amplification.

**Supplementary table 1**: primers used for qPCR

| Primer name | Sequence | T_a_ (ºC) | Primer efficiency (%) |
| --- | --- | --- | --- |
| RP11-204K16.1-F | 5’-CCCAAGATGCTGAAAGGAAAGAAAG-3’ | 58.8 | 93.51 |
| RP11-204K16.1-R | 5’-CTCAAACAGGGGATTCACCACTTC-3’ | 58.8 |  |
| XLOC_012542-F | 5’-GTGATTCAACAACCCTCCATCTCTG-3’ | 58.8 | 96.39 |
| XLOC_012542-R | 5’-TCACACACACCCATCCCATCC-3’ | 58.8 |  |
| RP4-609E1.2-F | 5’-TGTACCTCTTGTGGAGTCGTGTCTG-3' | 58.8 | 96.81 |
| RP4-609E1.2-R | 5’-AACTGGGATTCTGGCATCTTGAG-3' | 58.8 |  |
| AF015262.2-F | 5’-CCTGTGTCTTGACTGAGGGCTTATC-3' | 58.8 | 97.37 |
| AF015262.2-R | 5’-GCTTGCTGGTCTTGTCTGGCTC-3' | 58.8 |  |
| U6-F | 5’-CTCGCTTCGGCAGCACATATAC-3’ | 62.5 | 94.00 |
| U6-R | 5’-GGAACGCTTCACGAATTTGC-3’ | 62.5 |  |
| GAPDH-F | 5’-GGGAAACTGTGGCGTGATGG-3’ | 62.5 | 93.85 |
| GAPDH-R | 5’-TGGAGGAGTGGGTGTCGCTG-3’ | 62.5 |  |
| AC017078.1-F | 5’-GGTTGTGCGAGGACACCATAAAG-3’ | 58.8 | 99.17 |
| AC017078.1-R | 5’-TGCTGTACCCGTCCAATGTAGATG-3’ | 58.8 |  |
| XLOC_011152-F | 5’-AGGAAAGAGGGAAGATGGAGCAG-3’ | 58.8 | 95.87 |
| XLOC_011152-R | 5’-AATGAGGAAGGAGCGAAGGAGAG-3’ | 58.8 |  |
| RPS13 | Bio-Rad assay ID: qHsaCID0038672 | 60 | 97.00 |
